# Surgical management of pediatric urolithiasis

**DOI:** 10.4103/0970-1591.36718

**Published:** 2007

**Authors:** Shashi K. Mishra, A. Ganpule, T. Manohar, Mahesh R. Desai

**Affiliations:** Department of Urology, Muljibhai Patel Urological Hospital, Nadiad - 387 001, Gujarat, India

**Keywords:** Laparoscopy, percutaneous nephrolithotomy, pediatric, shock wave lithotripsy, urolithiasis, ureteroscopy, vesical

## Abstract

Pediatric urolithiasis poses a technical challenge to the urologist. A review of the recent literature on the subject was performed to highlight the various treatment modalities in the management of pediatric stones. A Medline search was used to identify manuscripts dealing with management options such as percutaneous nephrolithotomy, shock wave lithotripsy, ureteroscopy and cystolithotripsy in pediatric stone diseases. We also share our experience on the subject.

Shock wave lithotripsy should be the treatment modality for renal stone less than 1cm or < 150 mm^2^ and proximal non-impacted ureteric stone less than 1 cm with normal renal function, no infection and favorable anatomy. Indications for PCNL in children are large burden stone more than 2cm or more than 150mm^2^ with or without hydronephrosis, urosepsis and renal insufficiency, more than 1cm impacted upper ureteric stone, failure of SWL and significant volume of residual stones after open surgery. Shock wave lithotripsy can be offered for more soft (< 900 HU on CT scan) renal stones between 1-2cm. Primary vesical stone more than 1cm can be tackled with percutaneous cystolithomy or open cystolithotomy. Open renal stone surgery can be done for renal stones with associated structural abnormalities, large burden infective and staghorn stones, large impacted proximal ureteric stone. The role of laparoscopic surgery for stone disease in children still needs to be explored.

Urinary lithiasis affects between 5-10% of the humans during their lifetime, 2-3% of them are children.[1] Pediatric urolithiasis has wide epidemiologic variation in developed and the developing nations, with a prevalence of 1-5% to 5-15% respectively.[2] The literature on incidence, etiology and natural history of pediatric urolithiasis varies due to geographic, dietary and socioeconomic differences. Pediatric urolithiasis is known to be associated with urinary infection, anatomic and metabolic abnormalities. Management of stone disease in children necessitates complete stone clearance, eradication of urinary infection and appropriate correction of any underlying metabolic or anatomical abnormalities.[3] The purpose of this review article is to highlight the various treatment modalities in the management of pediatric stones. A Medline search was used to identify manuscripts dealing with management options such as percutaneous nephrolithotomy (PCNL), shock wave lithotripsy (SWL) and ureteroscopy (URS), cystolithotripsy and open surgery in pediatric stone diseases. We also share our experience on the subject.

## MANAGEMENT OF RENAL CALCULI

Management options for renal calculi are similar to those for adults. The majority of the stone disease in children can be managed with SWL, PCNL or a combination of treatment modalities. Open surgery is currently indicated in a few select cases. It is important to understand the effect of each treatment modality on the growth of the kidney. Stone location, composition, size; anatomy of collecting system; and presence of obstruction/infection are important factors in selecting the modality.

### Shock wave lithotripsy

Shock wave lithotripsy is currently the procedure of choice for treating most urinary stones in children. Shock wave lithotripsy should be the treatment modality for all renal stones less than 1 cm or < 150 mm^2^, soft renal stones (HU< 900 mm^2^ on CT scan) between 1 to 2 cm with normal renal function, no infection and favorable anatomy. However, the efficacy, need for ancillary procedures and treatment-related complications are not clearly defined as in the adult population. The theoretical long-term safety and bio effects of SWL on renal function and growth are debatable. Brinkman *et al.,*[4] noted no evidence of renal scarring, change in blood pressure or renal function loss following SWL, over 45 months in 63 children. Vlajkovic *et al.,*[5] calculated GFR in 84 children undergoing SWL using 99 m Tc-DTPA and noted a decrease in GFR immediately after SWL, which returned to pre-SWL level at three months. Kroovand *et al.,*[6] noted that radiation exposure during SWL treatment was comparable to other diagnostic radiographic procedures, such as VCUG.

Important considerations in SWL are stone burden, composition and ability of the distal urinary tract to successfully pass the fragments. Children pass stone fragments well and do not require stenting routinely. Newman *et al.,*[7] reported the first successful use of SWL in children in 1986. Gofrit *et al.,*[8] reported comparable clearance rates in children and adults after SWL for renal stones greater than 10mm. Technical modification in gantry or position is required to treat pediatric patients with SWL.[9]

Brinkman *et al.,*[4] reviewed results of HM3 second generation lithotripter and peizolith and found similar average stone-free rate of 80.8% and 74.5% respectively. Shorter duration of stone disease, greater stone fragility and lower impedance to shock wave might be possible reasons for better stone fragmentation. In children less than three and a half years of age, with stone size of 13mm, success rate was 66% and 86% in single and multiple sessions respectively. No complications were reported.[10]

Shock wave lithotripsy outcome for lower calyceal stones varies with lower pole anatomy. Tan *et al.,*[11] showed that infundibulopelvic angle and infundibular length significantly affected the stone-free rates with SWL for inferior calyceal stones. On the contrary, Onal *et al.,*[12] did not consider calyceal anatomy to have any significance. There is no uniform consensus regarding the anatomic factors that influence the clearance rate of calyceal stones after SWL, as different authors have studied various independent factors.

Management of pediatric staghorn calculus is technically challenging to the urologist. Al- Busaidy *et al.,*[13] found that in these patients, there was no difference in stone-free rates for stented and nonstented groups, however, complications were significantly higher in the latter group. Pre SWL stenting in staghorn calculi provided greater margin of safety and shortened the hospital stay. Shock wave lithotripsy in pediatric staghorn calculus has the shortcomings of multiple sessions, ancillary procedures and ureteral stenting. There is no comparative study of PCNL and SWL in pediatric staghorn calculi. Results of SWL in large stone burden are highly variable (33–83%). Wang *et al.,*[14] found that large maximal stone diameter (>12 mm), a high stone burden (>700 mm3), a high maximal stone density (>900HU) and stone shape (nonround/oval) on CT were significant predictors of SWL failure.

The cumulative risk of recurrence is higher in children as compared to adults. Afshar *et al.,*[15] reported that 34.5% fragments grew in size at mean follow-up of 48 months and a similar number of patients developed clinically significant symptoms. Nijman *et al.,*[16] have also reported that 33% children with small fragments had evidence of calculus growth at 24 months.

Shock wave lithotripsy is well tolerated with minimal morbidity. Minor complications such as bruising, ecchymosis and renal colic are reported in 11–50% cases. Authors treating large stone burdens have a reported steinstrasse rate of 1.9-5.4%.[17,18] Incidence of hematuria (40%) is less than that in adults. Ureteral obstruction or sepsis requiring stenting or percutaneous drainage may occur with large stone burden. The need for ancillary procedure is directly proportional to the size of stone treated.

We have performed SWL in 53 children (with a mean age of 6.2 + 4.4 years) using Dornier compact delta lithotripter (Dornier Medical Systems, Inc, Marietta, Georgia). General anesthesia was given to all pediatric patients at the start of SWL. Both ultrasound and fluoroscopy were used to localize and monitor the fragmentation. Shocks were started at Level one (10 kilovolts) and progressed to Level two (11.5 kilovolts) after 100 shocks. The intensity was increased to higher levels only if the desired fragmentation was not visible with fluoroscopy and ultrasound. In children, the power settings rarely exceeded Level three (12.75 kilovolts). The shocks were given at a frequency of 60. Procedure was terminated after complete fragmentation was noted on fluoroscopy and ultrasound. Number of shocks given never exceeded 1500. The mean stone length was 1.09 + 0.4 cm. The mean shocks required per session were 982 + 492. The mean intensity of the shocks was 11.81 + 0.5 kilovolts. The mean number of sessions required was 1.09 + 0.3. Adequate fragmentation was achieved in all. We feel that complete clearance should be achieved with minimal number of shocks, energy and need for ancillary procedures. Clinically insignificant residual fragments (CIRF) can be a source of recurrent stone formation and hence not considered as success. Overall complete clearance was achieved in 42 (79.2%) renal units at the end of three months. Seven (13.2%) patients had CIRF which was being conservatively followed. Ancillary procedures were required in four (7.5%) renal units, which included PCNL in three and ureteroscopy in one child.

### Percutaneous nephrolithotomy

Since the first pediatric series reported by Woodside and associates in 1985,[19] PCNL has become an established technique in children as monotherapy or as part of a multimodal approach for children with large stone burdens. The reluctance to perform PCNL in children was due to concern regarding long-term renal damage, small kidney size, relatively large instruments, radiation exposure and risk of major complications such as bleeding.

Studies demonstrate minimal scarring and insignificant loss of renal function after PCNL.[20] Dawaba *et al.,*[21] reported no renal scarring in 65 patients on long-term follow-up. Earlier studies with PCNL in children described the use of adult size instruments. Desai *et al.,*[22] showed that intraoperative hemorrhage during PCNL in children is related to the caliber and number of tracts and emphasized the need of technical modifications. Ultrasound-guided peripheral calyceal puncture and restricting tract size to 22F are important factors in reducing the blood loss. Multiple tracts increase the hemoglobin drop but are not associated with an increased risk of complications (bleeding, postoperative infection and prolonged urinary leak). Zeren *et al.,*[23] also showed significant association of intraoperative bleeding with operative time, stone burden and sheath size.

Improvement in technology and miniaturization of instruments, with availability of more efficient energy sources for intracorporeal lithotripsy has revolutionized endourological procedures in children. Helal *et al.,*[24] reported using 15Fr access sheath in a two-year-old child. Jackman *et al.,*[25] described an 11Fr access sheath for pediatric PCNL.

With the availability of holmium-Yttrium Almunium Garnet (Ho: YAG) laser, smaller pneumatic lithoclast and ultrasound probes, PCNL can be performed using smaller nephroscopes. We designed a smaller lithoclast probe with suction for use through a pediatric nephroscope and found it highly effective and safe in children. Various studies have demonstrated the safety of Ho: YAG laser in children. Ultrasound-guided puncture is a good alternative to fluoroscopy and has the advantage of avoiding radiation and preventing visceral injury.[26]

Complications are similar to adults. Intraoperative bleeding requiring blood transfusion, injury to the pelvicaliceal system and sepsis are major concerns with PCNL in children. Kroovand *et al.,*[27] proposed a two-session approach to minimize bleeding; initially establishing percutaneous tract and a second session for calculus clearance.

**Figure 1:**
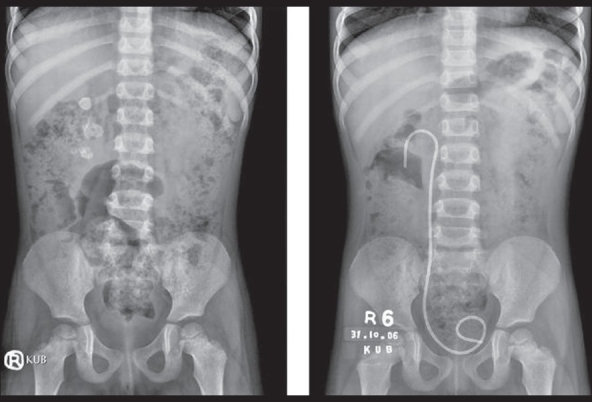
Four years old child with Right complex calculi (left), treated with PCNL complete clearance achieved (right)

Indications for PCNL in children are similar to those in adults and include large burden stone more than 2cm, hard renal stone (> 900HU on CT scan) between 1 to 2cm, significant renal obstruction, urinary infection, failure of SWL and significant volume of residual stones after open surgery. We have performed PCNL in 222 renal units in children (mean age 8.9 ± 3.9 years) from 1997 till date. Mean stone bulk was 335.6±122.6 mm^3^ (range: 94-989) with 130 complex calculi. In our earlier published data the stone clearance rate was 89.8%. With ancillary procedures (SWL), stone clearance increased to 96% at three months.[28] For children less than five years old, for staghorn and complex calyceal calculi, we achieved stone-free rate of 86%, with mean hemoglobin drop of 2.2 ± 0.95 g/dL and mean hospital stay of 3.5 days[29] [Figure 1].

**Figure 2:**
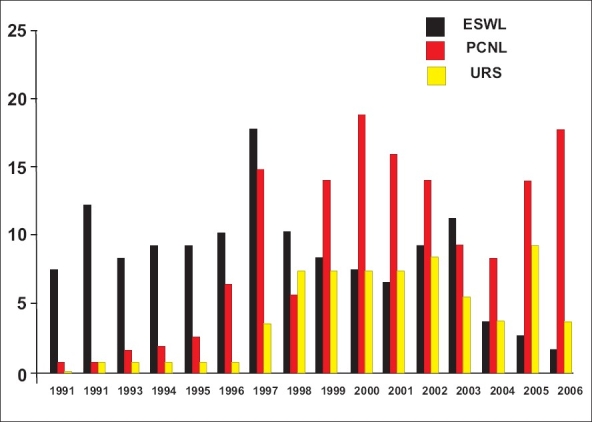
Trend of various pediatric urolithiasis procedures over the years, at our institute

Percutaneous nephrolithotomy and SWL are safe and efficacious in managing pediatric stones of 1-2cm; however, the choice should be tailored to the three-dimensional stone size and composition, using 3-D CT scan.[14] When we analyze the trends of SWL, PCNL and ureteroscopy done for pediatric urolithiasis over the years [Figure 2], PCNL has become the mainstay, gradually replacing SWL. The probable reason for this may be that more stones are presenting with a size between 1-2 cm. We routinely do two-dimensional measurement of the stone burden by plain radiograph that may not give accurate measurement of the stone bulk. Therefore it is important to do 3-D CT to know the exact stone size and to estimate Hounsfield unit to predict stone density.

Recurrence is a major problem as follow-up is not assured due to poor socioeconomics. In this scenario, SWL has higher retreatment rates requiring more ancillary procedures, thus defeating the purpose of giving the patient complete stone clearance with minimal morbidity and a single hospital stay.[30] However, one must not forget that PCNL is a potentially morbid procedure and can be accompanied by life-threatening bleeding and sepsis. Children are fragile as body reserves are less. Hence, the learning curve has to be taken into consideration, before starting pediatric PCNL. We stage the procedure in select cases such as nondilated system, associated infection and large stone burden to decrease complications and increase the success rate of the procedure.[29] Restricting the percutaneous tract to 20- 22Fr compared to the conventional 30Fr conventional tract may significantly reduce the morbidity.

### Laparoscopic surgery

Laparoscopic renal surgery is still not widely performed by pediatric urologists due to higher operative time, logistic support, lack of indications and sufficient surgeon experience. Laparoscopic retroperitoneal surgery has a definite role in the management of patients requiring open surgery for calculus disease but indications in pediatric patients are not well defined. Casale *et al.,*[31] reported experience with transperitoneal laparoscopic pyelolithotomy in pediatric patients in whom percutaneous renal access failed and the stone burden warranted open intervention. Stones in the renal pelvis were removed with rigid graspers under direct laparoscopic vision. A flexible cystoscope was used for caliceal stones. The renal pelvis was sutured intracorporeally constructing watertight anastomosis. There was significant operative time, however, it still had the advantages of minimally invasive surgery. Laparoscopic assisted percutaneous transperitoneal nephrolithotomy appears to be a simple and suitable minimally invasive treatment of the stone-holding pelvic dystopic kidney. Gaur *et al.*[32] successfully performed retroperitoneal laparoscopic ureterolithotomy in five patients with calculi impacted in the upper and middle ureter. Interestingly, all the patients were discharged after 24h.

## MANAGEMENT OF URETERAL CALCULI

Van savage *et al.,*[33] noted that most stones less than 3 mm in diameter in the distal ureter of children would pass spontaneously. Stones 4 mm or greater in diameter are likely to require treatment. Most of the ureteric stones can be managed with SWL or URS.

### SWL

Stone-free rates with SWL vary from 75-100% depending on the size of the stone. Landau *et al*.[34] found that 100% of patients with stones less than 10mm were stone-free regardless of the location. Stone-free rates were 67% in stones larger than 10 mm following a single SWL session. Shock wave lithotripsy may require ureteral stenting in a large population of children with ureteral calculi, either to aid localization or stone clearance. We did four SWLs for upper ureteric stones with a success rate of 75%. One patient required ancillary procedure (URS) for strainstrasse. De Dominics *et al.,*[35] found significantly lower efficiency quotient for treating distal ureteric calculi for SWL than retrograde ureteroscopic stone removal in the pediatric age group.

### Ureteroscopy

With the advent of smaller instruments and laser lithotripsy, URS for management of pediatric urolithiasis has become more common. With the availability of 4.5 and 6Fr semi-rigid ureteroscopes and a 6.9Fr flexible ureterorenoscope with Ho: YAG laser energy source, instrument-related complications are uncommon. Ho-YAG laser fibers are small and flexible with a short depth of penetration (0.4 mm), allowing it to be used safely with pediatric endoscopes. Further, laser fragmentation produces 2-3 mm fragments that can pass very easily down the ureter. Most series have employed ureteral stent placement, following ureteroscopic lithotripsy in pediatric patients. We reported successful use of supine antegrade flexible ureteroscopy in treating impacted upper ureteric calculi in a six-year-old pediatric patient.[36]

It has been shown that ureteral dilatation does not increase the risk of stricture and significant vesicoureteric reflux (VUR). Caione *et al.,*[37] reported no post ureteroscopy VUR in seven children after rigid ureteroscopy. Shepherd *et al.,*[38] have shown that dilating the ureter up to 12Fr did not result in VUR postoperatively. Voiding cystograms done on pediatric patients after ureteroscopic procedures have shown the incidence of low-grade VUR to be as high as 15%.[39,40] The most common complication following pediatric ureteroscopic lithotripsy is postoperative urosepsis and pain. Reported incidence of ureteral perforation in published studies is 1.4%, similar to that seen in adult patients.[41]

Ureteroscopy may provide more efficient stone clearance and hence should be preferred for distal ureteral stones, larger stones and impacted stones. We have performed URS in 86 patients (mean range 1–17 years, size 4-16mm). Ureteroscopy was possible with 6Fr semi-rigid ureteroscope in 81 while 6.8/7.5Fr flexible ureteroscope was used in five patients. Seventy-two (83.7%) patients had mid or lower ureteric stones and 14 (16.2%) patients had upper ureteric stones. Foty-eight (55.8%) patients required ureteric dilatation. Double J stent was placed postoperatively in 36 (41.8%). The procedure was successfully completed in all except one who required simultaneous antegrade flexible URS. Mean hospital stay was three days.

## MANAGEMENT OF BLADDER CALCULI

Vesical stones in the pediatric age group in India, often present with a large stone burden. Vesical calculi can be managed by transurethral or percutaneous suprapubic lithotripsy. In children, especially in boys, because of the small caliber penile urethra and concerns about iatrogenic urethral stricture, transurethral cystolithotripsy may be more difficult. It is safe if stone burden is less than 1cm. Percutaneous cystolithotomy (PCCL) is a safe alternative with low morbidity and complication rate for large burden vesical stone.[42–45] Percutaneous cystolithotomy has been performed safely for bladder stones up to 5cm in size. Using the percutaneous suprapubic approach, a 26F nephroscope can be introduced into the bladder without urethral injury. The large and hard stones can be disintegrated and removed in large fragments, so that the intervention can be performed quickly. The technique is also more advantageous than open surgery with regard to cosmetic outcome and length of the hospital stay. Open cystolithotomy has the inherent problems of a long scar, prolonged catheterization, extended hospitalization and risk of infection. It still is recommended for multiple bladder calculi and even for solitary vesical stone of any size where PCCL is not available.

We have performed 81 bladder stone surgeries between 1995 and 2006. The mean age of the patients was 6.4 ± 2.3 years, range 1-15 years. The surgeries performed were cystolithotripsy (n=60), PCCL (n=13) and open cystolithotomy (n=8). We have used pneumatic, ultrasound and laser energy for stone fragmentation. In the pediatric age group (less than five years), a 7.5Fr ureteroscope/pediatric cystoscope was used.

## MANAGEMENT OF URETHRAL STONE

Sometimes small stones migrate from the upper urinary tract into the bladder and then are ejected out per urethra with the urinary stream. Infrequently, during passage through the urethra the calculus gets impacted even when there is no distal organic obstruction. The management of such recently impacted calculus varies according to the site and nature of the calculus. The methods to deal with the situation are to bring out the stone by forward milking or external urethrotomy if situated in the anterior urethra. The former procedure is quite traumatizing to the urethral wall and should be restricted to only smooth contour stone in presence of non-obstructed urethra. External urethrotomy even as a primary procedure is best avoided in penile urethra. Stone can also be removed with gentle pulling with artery forceps if lodged at the submeatus under proper anesthesia. In the case of posterior urethral stone, push back by applying vigorous per urethral xylocaine jelly and single attempt of per urethral catheterization(PUC) is worth trying. If unsuccessful, it is advisable to do suprapubic cystostomy (SPC) to prevent further urethral damage. After initial decompression of bladder with PUC / SPC, later endoscopic removal of the stone can be undertaken.

## ROLE OF OPEN STONE SURGERY

Open stone surgery should be offered in situations where it is at least a viable and reasonable alternative to less invasive modalities. The pros and cons of the treatment should be explained in an unbiased manner to the attendants to effectively perform and implement this form of treatment if chosen.[46] Sakkas *et al.,*[47] estimated urinary levels of N-acetyl-glycosaminidase (NAG), a sensitive marker of renal tubular damage and found significant renal functional damage with open stone surgery compared with SWL and PCNL post treatment. Open stone surgery can be offered in the presence of associated structural abnormalities (e.g. PUJ obstruction) or large burden infective and staghorn stones, large proximal ureteric stones and large bladder calculus. In addition, it can be applied successfully at places where minimal invasive treatment modality is unavailable and parents’ preference is for open surgery. In some developing countries, reported incidence (14%) is higher than that reported in industrialized countries (0.3-5.4%).[48] The poor socioeconomics favors cost and an assured stone-free status more importance than incision or incision-free surgery. Moreover, in children, healing is rapid and less complicated than in adults and thus the convalescence period is short with open surgery.[48] Zargooshi et al. achieved overall stone-free rate of 95.4% in 296 children with 100% for single stones. They concluded that open stone surgery was safe, cost-effective, associated with excellent patient acceptance, low morbidity and good stone-free rates. Assimos *et al.*,[49] reported successful pediatric anatrophic nephrolithotomy in 10 of 11 patients with minimal morbidity, but with high recurrence. Open surgery, which is nowadays being replaced with laparoscopic techniques, is generally indicated for failed endourological procedures for upper ureteric stone, particularly in centers that do not have flexible ureteroscopy or laser lithotriptor and in patients with larger stones (>3 cm).[50] Open surgical removal of primary bladder stone in children is still the mainstay of therapy in spite of miniaturization of endoscopes. Mahran *et al.*, compared the efficacy of open cystolithomy and cystolitholapaxy in pediatric patients with primary bladder stones. The operative time was similar in the two groups. The hospital stay was significantly less after endourologic procedures than after open surgery. However, there were significantly more complications in the endourologic procedures.[51]

## CONCLUSION

Pediatric urolithiasis poses a technical challenge to the urologist. Aims of the management should be complete clearance of stones, preservation of renal function and prevention of recurrence. Despite the consensus of SWL being the initial treatment of choice for most stones in pediatric patients, there are certain indications for other modalities as well. With improvement in instrumentation and technology, endoscopic management has become safe and effective. Percutaneous nephrolithotomy and SWL are safe and efficacious in managing pediatric stones of 1-2cm. Indications for PCNL in children are large stone burden, significant renal obstruction and renal infection. Ureteroscopy provides efficient stone clearance in mid and lower ureteric stones. Transurethral cystolithotripsy is generally avoided in pediatric patients, but is feasible in single vesical stone less than 1cm. Percutaneous cystolithotomy or open cystolithotomy is generally the alternative for pediatric vesical stones.
